# Determinants of cyberchondria in Tunisian university students

**DOI:** 10.1192/j.eurpsy.2026.10785

**Published:** 2026-07-31

**Authors:** H. Guidara, I. Chaari, Z. Hadrich, M. Kessentini, W. Abid, E. Maaloul, A. Kammoun, F. Charfeddine, L. Aribi, N. Messedi, J. Aloulou

**Affiliations:** 1 Psychiatric department “B”, Hedi Chaker University Hospital; 2 Higher institute of nursing sciences of Sfax, Sfax, Tunisia

## Abstract

**Introduction:**

Cyberchondria is frequent among young people and influenced by multiple determinants. Identifying these risk factors is essential to prevention. However, data from the Maghreb region are limited.

**Objectives:**

To explore sociodemographic, academic, and psychiatric predictors of cyberchondria in Tunisian students.

**Methods:**

We conducted a cross-sectional online survey among university students in Sfax (Tunisia) between March and April 2025. Validated instruments were used: Cyberchondria Severity Scale-15 (CSS-15), Short form of Health Anxiety Inventory (SHAI), Internet addiction test (IAT), and eHealth literacy scale (eHEALS). Students completed also sociodemographic and psychiatric questionnaires. Statistical analyses included Mann–Whitney U, Kruskal–Wallis, and Spearman correlations.

**Results:**

A total of 283 Tunisian students were analyzed (117 females, 166 males). Cyberchondria scores were higher in females (p<0.001) and in first- and fourth-year students (p<0.001). Family psychiatric history (p<0.001) and personal psychiatric history (p=0.005) were associated with elevated scores. Cyberchondria correlated positively with Internet addiction (ρ=0.22, p<0.001) and negatively with eHealth literacy (ρ=−0.14, p=0.017).

**Conclusions:**

Among Tunisian university students, cyberchondria is shaped by gender, stage of training, psychiatric vulnerability, and digital behaviors. Preventive strategies should target these risk groups and promote healthier Internet use.

**Disclosure of Interest:**

None Declared

